# Beneficial Effects of Dietary Nitrite on a Model of Nonalcoholic Steatohepatitis Induced by High-Fat/High-Cholesterol Diets in SHRSP5/Dmcr Rats: A Preliminary Study

**DOI:** 10.3390/ijms23062931

**Published:** 2022-03-08

**Authors:** Kunihiro Sonoda, Yuka Kono, Kazuya Kitamori, Kazuo Ohtake, Sachiko Shiba, Keizo Kasono, Jun Kobayashi

**Affiliations:** 1 Department of Food and Nutritional Environment, College of Human Life and Environment, Kinjo Gakuin University, Nagoya 463-8521, Japan; u2014002@kinjo-u.ac.jp (Y.K.); kitamori@kinjo-u.ac.jp (K.K.); 2 Division of Physiology, School of Pharmaceutical Sciences, Faculty of Pharmaceutical Science, Josai University, Saitama 350-0248, Japan; kazuo@josai.ac.jp (K.O.); s-shiba@josai.ac.jp (S.S.); kasono@josai.ac.jp (K.K.); 3 Division of Pathophysiology, Department of Clinical Dietetics and Human Nutrition, Faculty of Pharmaceutical Science, Josai University, Saitama 350-0248, Japan; junkoba@josai.ac.jp

**Keywords:** nonalcoholic fatty liver disease, nonalcoholic steatohepatitis, nitric oxide, nitrite, nitrate, angiotensin II, angiotensin II type 1 receptor, captopril, nicotinamide adenine dinucleotide phosphate oxidase, endothelial dysfunction

## Abstract

Nonalcoholic steatohepatitis (NASH) is a chronic liver disease that leads to liver cirrhosis and hepatocellular carcinoma. Endothelial dysfunction caused by hepatic lipotoxicity is an underlying NASH pathology observed in the liver and the cardiovascular system. Here, we evaluated the effect of dietary nitrite on a rat NASH model. Stroke-prone, spontaneously hypertensive 5/Dmcr rats were fed a high-fat/high-cholesterol diet to develop the NASH model, with nitrite or captopril (100 mg/L, each) supplementation in drinking water for 8 weeks. The effects of nitrite and captopril were evaluated using immunohistochemical analyses of the liver and heart tissues. Dietary nitrite suppressed liver fibrosis in the rats by reducing oxidative stress, as measured using the protein levels of nicotinamide adenine dinucleotide phosphate oxidase components and inflammatory cell accumulation in the liver. Nitrite lowered the blood pressure in hypertensive NASH rats and suppressed left ventricular chamber enlargement. Similar therapeutic effects were observed in a captopril-treated rat NASH model, suggesting the possibility of a common signaling pathway through which nitrite and captopril improve NASH pathology. In conclusion, dietary nitrite attenuates the development of NASH with cardiovascular involvement in rats and provides an alternative NASH therapeutic strategy.

## 1. Introduction

Nonalcoholic fatty liver disease (NAFLD) is characterized by the accumulation of triglycerides in the liver tissues of patients without a history of excessive alcohol consumption. NAFLD can be categorized as simple liver steatosis (nonalcoholic fatty liver: NAFL) or nonalcoholic steatohepatitis (NASH) with intralobular inflammation, ballooning degeneration of hepatocytes, and fibrosis [1]. Excess hepatic fat deposition in patients with NAFLD is associated with an increased risk of diabetes, hypertension, and cardiovascular events, which are caused by lipotoxic inflammatory mediators targeting the vascular endothelium of the liver and cardiovascular system [2]. Apart from lifestyle modifications through diet and exercise, currently, no specific treatments for NAFLD are known.

Cordero-Herrera et al. recently reported that dietary nitrate/nitrite administration prevented the development of NAFL by activating AMP-activated protein kinase and inhibiting nicotinamide adenine dinucleotide phosphate (NADPH) oxidase, which reduced hepatic lipid accumulation, in a mouse model of metabolic syndrome resulting from the chronic administration of a high-fat diet and a nitric oxide synthase (NOS) inhibitor (*N*ω-Nitro-L-arginine methyl ester hydrochloride) [3]. Liu et al. also reported that dietary nitrate attenuates the progression of liver steatosis in another model of ApoE−/− mice fed a high-fat diet [4]. However, there have been few reports concerning the effect of dietary nitrate/nitrite on the NASH model. NASH is more severe than NAFL; it is currently considered the most frequent cause of chronic liver impairment in developed countries and is also a major causative factor in liver cirrhosis and hepatocellular carcinoma development [5]. Regarding animal experiments used to study NASH pathology, a stroke-prone (SP) spontaneously hypertensive 5/Dmcr (SHRSP5/Dmcr) rat model, established from a substrain of SP spontaneously hypertensive rats fed a high-fat/high-cholesterol (HFC) diet, was first reported in 2012 as a novel rat model for the development of hepatic lesions similar to those observed in human NASH pathology [6,7]. This model also showed the simultaneous development of hypertension because NASH exerts a pathological influence on the cardiovascular system [8]. Recent reports from Zhang et al. [9] and Toblli et al. [10] show that angiotensin-converting enzyme (ACE) inhibitors prevent not only liver-related events in NAFLD in humans but also fatty liver and fibrosis in an obese zucker NASH rat model. These results may indicate that hepatic and cardiovascular endothelial dysfunction potentially represents at least one of the common underlying etiologies of NASH pathology [2,8]. NO-mediated signaling, including that induced through dietary and pharmacological means, could possibly provide preventive and therapeutic options for this disease.

NO is generated through the enzymatic L-arginine/NOS pathway and is an important mediator in regulating vascular tone, neurotransmitters, and host defense [11]. In addition to enzymatic NO generation, the enterosalivary nitrate–nitrite–NO pathway is an alternative for gastric and systemic NO generation, particularly when endogenous NO generation is impaired [11]. Dietary and enterosalivary nitrate are reduced to nitrite by commensal bacteria present in the oral cavity. Following deglutition to the stomach, nitrite is reduced to nitrous acid by H^+^ present in gastric juice, which decomposes to NO and other nitrogen oxides (such as dinitrogen trioxide [N_2_O_3_] and NO_2_). N_2_O_3_ can donate NO^+^ and transnitrosylates, the key signaling thiol proteins involved in gastric and systemic NO-mediated physiology [11]. Erythrocytes also play an important role in the systemic transfer of NO. In addition to their role in transporting and excretion of the end-products of NO oxidation (nitrate/nitrite) during blood circulation from arteries to veins, erythrocytes reduce nitrite to NO using the reductase activity of deoxyhemoglobin [12,13]. This helps maintain the supply of NO in the form of NO and protein S-nitrosothiols for local and systemic NO-mediated activities.

Because limited research has been conducted on the effect of dietary nitrate/nitrite on NASH with cardiovascular involvement, we attempted to evaluate the effects of dietary nitrite supplementation on NASH pathology in this rat model to determine the molecular mechanism underlying the action of nitrite, particularly compared with that of the ACE inhibitor, which has been demonstrated to have a preventive effect on the development of NASH pathology [10]. We show that dietary nitrite and the ACE inhibitor captopril may be therapeutically involved in the angiotensin II type 1 receptor (Ang II -AT1R) pathway and propose a therapeutic strategy for preventing NASH development.

## 2. Results

### 2.1. Effects of Nitrite and Captopril on the Body, Liver, and Heart Weights and Blood Pressure of the Rat NASH Model

**Figure 1:**
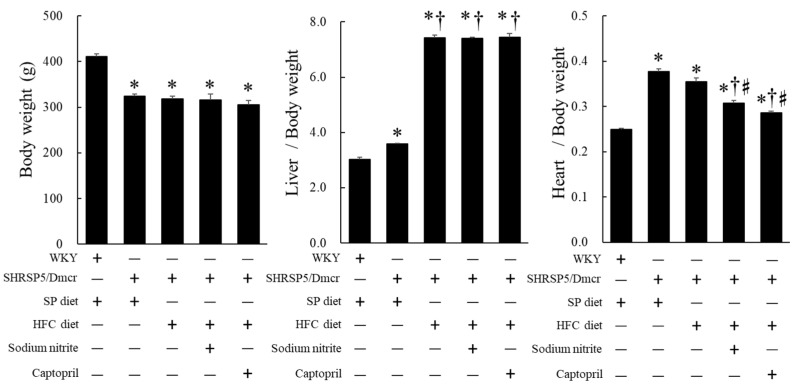
Effects of nitrite and captopril supplementation on body, liver, and heart weights in the rat nonalcoholic steatohepatitis model. Values represent mean ± SE (*n* = 6), * *p* < 0.05 vs. WKY + SP diet group, ^†^ *p* < 0.05 vs. SHRSP5/Dmcr + SP diet group, ^#^ *p* < 0.05 vs. SHRSP5/Dmcr + HFC diet group. SP diet, stroke-prone diet; HFC diet, high-fat/high-cholesterol diet; WKY, Wistar Kyoto rat.

The body weight (BW) of SHRSP5/Dmcr rats decreased compared to that of control Wistar Kyoto (WKY) rats fed an SP diet; however, there was no significant difference among the BWs of SHRSP5/Dmcr rats, regardless of the diet or nitrite/captopril supplementation via drinking water. The liver weight corrected by the BW increased in SHRSP5/Dmcr rats fed the SP diet compared to that of rats fed the SP diet, and further increased in SHRSP5/Dmcr rats fed the HFC diet compared to that of rats fed the SP diet. Nitrite and captopril exerted no significant effect on the liver weights corrected by BW of HFC diet-fed SHRSP5/Dmcr rats. The heart weight corrected by BW of SHRSP5/Dmcr rats fed the SP or HFC diet increased compared to that of control WKY rats fed the SP diet, whereas the supplementation of HFC diet-fed SHRSP5/Dmcr rats with nitrite and captopril in drinking water (100 mg/L each for 8 weeks) significantly decreased the heart weight corrected by the BW (Figure 1).

**Figure 2:**
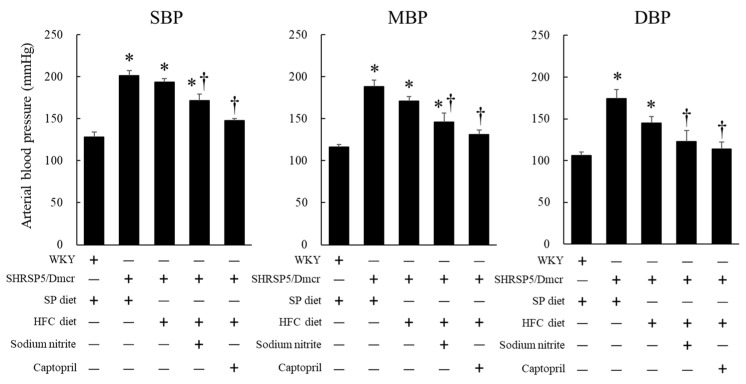
Effects of nitrite and captopril on blood pressure in the rat nonalcoholic steatohepatitis model. Values represent mean ± SE (*n* = 6), * *p* < 0.05, vs. the WKY + SP diet group, ^†^ *p* < 0.05, vs. the SHRSP5/Dmcr + SP diet group. SBP, systolic blood pressure; MBP, mean blood pressure; DBP, diastolic blood pressure.

The systolic, mean, and diastolic blood pressures of SHRSP5/Dmcr rats fed the SP and HFC diets were significantly elevated compared to those of control WKY rats fed the SP diet. There was a downward but not significant trend in the blood pressures (systolic, mean, and diastolic) of the rats in the SHRSP5/Dmcr fed HFC diet group fed a supplementation of nitrite (or captopril) (100 mg/L each for 8 weeks) compared to those of the rats in the HRSP5/Dmcr fed HFC diet group (Figure 2).

### 2.2. Effects of Nitrite and Captopril on Liver Steatosis in the Rat NASH Model

**Figure 3:**
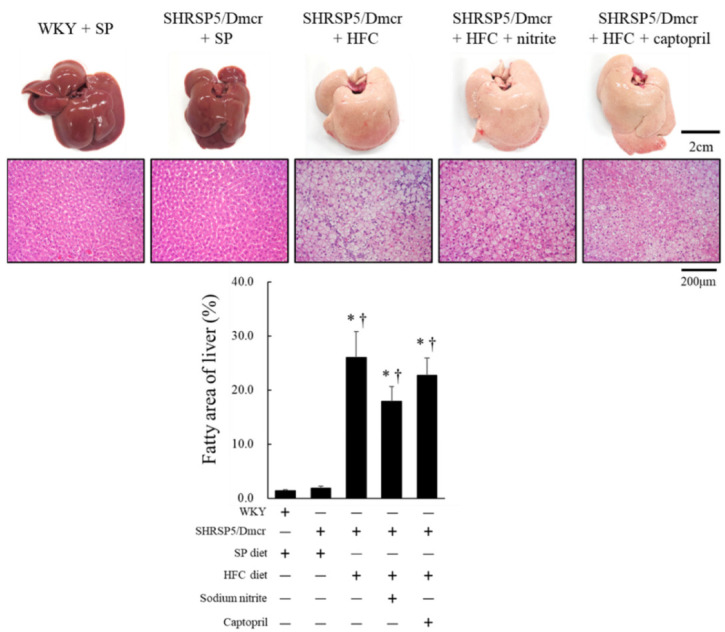
Macroscopic appearance and histological analysis (HE staining) of liver in WKY and SHRSP5/Dmcr rats fed SP diets and nonalcoholic steatohepatitis model rats treated with or without nitrite and captopril. Values represent mean ± SE (*n* = 6), * *p* < 0.05, vs. the WKY + SP diet group, ^†^ *p* < 0.05, vs. the SHRSP5/Dmcr + SP diet group.

Liver fatty areas stained using hematoxylin and eosin (HE) were evaluated in the rat NASH model (Figure 3). There is a clear difference in the macroscopic and microscopic appearance of the liver between the groups (WKY and SHRSP5/Dmcr) fed the SP diet, and the SHRSP5/Dmcr rat groups fed the HFC diet, with and without dietary nitrite and captopril. Compared to the WKY and SHRSP5/Dmcr-fed SP diet rats, hepatic lipid deposition expressed by fatty area significantly increased in the SHRSP5/Dmcr rats fed the HFC diet both with and without dietary nitrite or captopril. An improvement in liver steatosis was observed in the rats supplemented with dietary nitrite and captopril, but this was not statistically significant in the groups of SHRSP5/Dmcr rats fed the HFC diet.

Consistent with the effects of nitrite and captopril on liver steatosis in the rat NASH model, dietary nitrite and captopril supplementation had no significant effect on lowering the increased plasma levels of aspartate aminotransferase (AST) or alanine aminotransferase (ALT) in the rat NASH models (Figure S1).

### 2.3. Effects of Nitrite and Captopril on Liver Inflammation and Fibrosis in the Rat NASH Model

In the present study, inflammatory cells infiltrated in the liver were morphologically identified in the hepatic tissue specimens stained using HE. They were identified as lymphocytes, macrophages, and granulocytes in the rat NASH model (Figure S2, left). Immunohistochemical staining was also performed for further investigation.

**Figure 4:**
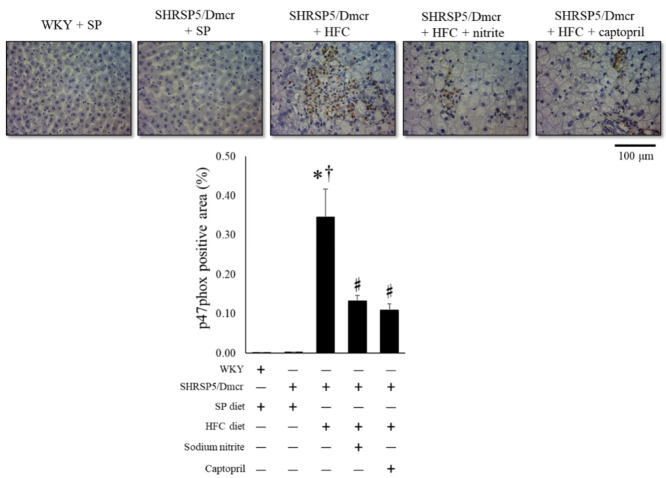
Immunohistochemical analysis of p47phox-positive area in the hepatic tissues of the rat nonalcoholic steatohepatitis model treated with nitrite and captopril. Results are expressed as mean ± SE (*n* = 6), Values represent mean ± SE (*n* = 6), * *p* < 0.05 vs. WKY + SP diet group, ^†^ *p* < 0.05 vs. SHRSP5/Dmcr + SP diet group, ^♯^ *p* < 0.05 vs. SHRSP5/Dmcr + HFC diet group.

The immunohistochemical expression of the NADPH oxidase component p47phox, which induces the generation of reactive oxygen species (ROS) and causes subsequent inflammatory cell-mediated hepatic injury and fibrosis [14], increased in SHRSP5/Dmcr rats fed the HFC diet compared to that in rats fed the SP diet. However, p47phox expression in the liver decreased significantly in response to nitrite and captopril supplementation (Figure 4).

**Figure 5:**
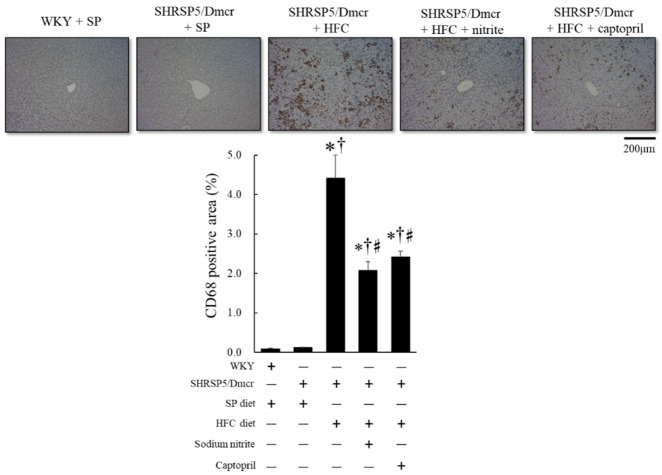
Immunohistochemical analysis of CD68-positive area in the hepatic tissues of the rat nonalcoholic steatohepatitis model and the effects of nitrite and captopril on the inflammatory response in the liver. Values represent mean ± SE (*n* = 6), * *p* < 0.05 vs. WKY + SP diet group, ^†^ *p* < 0.05 vs. SHRSP5/Dmcr + SP diet group, ^♯^ *p* < 0.05 vs. SHRSP5/Dmcr + HFC diet group.

To identify the inflammatory cells expressing p47phox (Figure S2, middle), CD68 immunostaining specific to macrophages was performed. There were many CD68 immunostaining positive cells in the same liver tissue section as there were where p47phox was positive, suggesting that most of the cells expressing p47phox are macrophages (Figure S2, right). The accumulation of macrophages, including activated Kupffer cells (as indicated by the immunohistochemical expression of CD68 in the liver), was enhanced in SHRSP5/Dmcr rats fed the HFC diet compared to both control WKY and SHRSP5/Dmcr rats fed the SP diet; however, it reduced significantly in response to nitrite and captopril supplementation (Figure 5).

**Figure 6:**
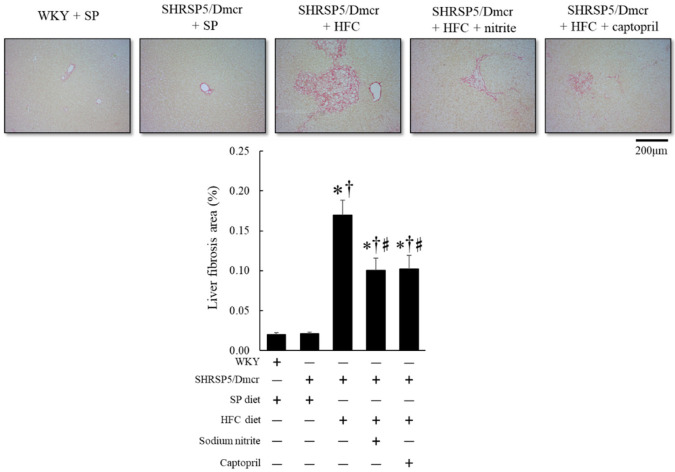
Histological analysis of liver fibrosis (Sirius Red stain) in the rat nonalcoholic steatohepatitis model treated with nitrite and captopril. Values represent mean ± SE (*n* = 6), * *p* < 0.05 vs. WKY + SP diet group, ^†^ *p* < 0.05 vs. SHRSP5/Dmcr + SP diet group, ^♯^ *p* < 0.05 vs. SHRSP5/Dmcr + HFC diet group.

Consistent with the therapeutic effect of nitrite and captopril on hepatic inflammation, as shown in Figure 4 and Figure 5, liver fibrosis in SHRSP5/Dmcr rats, caused by the intake of the HFC diet decreased significantly in response to nitrite and captopril supplementation, as observed using Sirius Red staining (Figure 6).

### 2.4. Effects of Nitrite and Captopril Supplementation on Dilated Left Ventricle (LV) and Coronary Perivascular Fibrosis in the NASH Model

**Figure 7:**
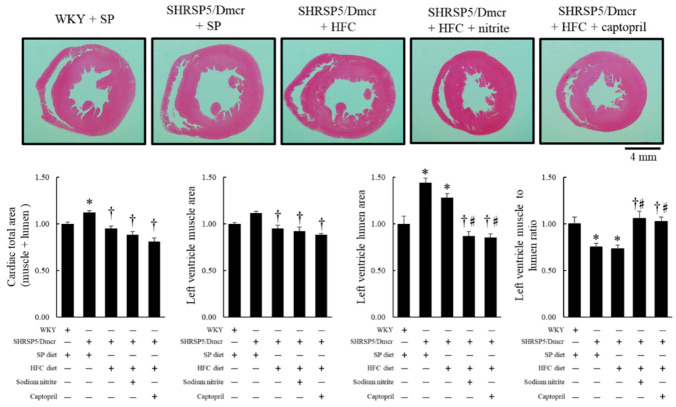
Gross photograph of midventricular short-axis section and quantitative analysis of left ventricular muscle area and chamber size following treatment with nitrite and captopril. Values represent mean ± SE (*n* = 6), * *p* < 0.05 vs. WKY + SP diet group, ^†^ *p* < 0.05 vs. SHRSP5/Dmcr + SP diet group, ^♯^ *p* < 0.05 vs. SHRSP5/Dmcr + HFC diet group.

Concerning the protective effects of dietary nitrite and captopril on cardiovascular involvement in the rat NASH model, SHRSP5/Dmcr rats fed SP and HFC diets developed LV chamber enlargement (in the LV lumen area), with a lower LV muscle/chamber area ratio than that of control WKY rats fed an SP diet; however, the effect was reversed by nitrite and captopril supplementation in drinking water (Figure 7).

In a subgroup experiment (supplementation with 500 mg/L nitrite in drinking water for 4 weeks), perivascular fibrosis of the coronary artery increased in SHRSP5/Dmcr rats fed the HFC diet compared to that in rats fed the SP diet, though nitrite supplementation reduced HFC diet-induced coronary artery fibrosis (Figure S3) [8]. The mRNA levels of brain natriuretic peptide (BNP), a heart failure marker synthesized in the LV in response to pressure and volume overload [15], increased in the myocardium of SHRSP5/Dmcr rats fed an HFC diet; however, it decreased in response to nitrite supplementation in drinking water (500 mg/L in drinking water administered for 4 weeks). Meanwhile, there were no significant differences in the mRNA levels of atrial natriuretic peptide (ANP) among SHRSP5/Dmcr rats, regardless of the diet and nitrite supplementation (Figure S4).

### 2.5. Effects of Nitrite and Captopril Supplementation on the Plasma Levels of Nitrite and Nitrate in the NASH Model

**Figure 8:**
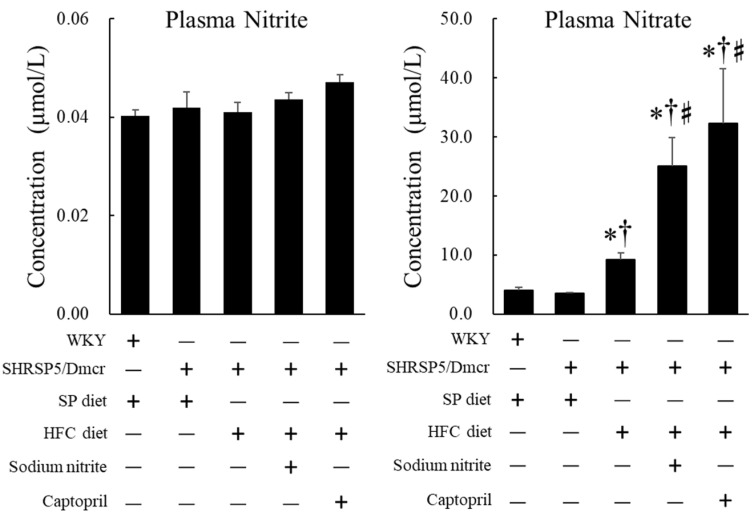
Effects of nitrite and captopril supplementation on the plasma levels of NOx in the rat nonalcoholic steatohepatitis model. Values represent mean ± SE (*n* = 6), * *p* < 0.05 vs. WKY + SP diet group, ^†^ *p* < 0.05 vs. SHRSP5/Dmcr + SP diet group, ^♯^ *p* < 0.05 vs. SHRSP5/Dmcr + HFC diet group. NOx, nitrite, and nitrate.

There were no significant differences in the plasma nitrite levels between WKY rats and SHRSP5/Dmcr rats, regardless of the diet or nitrite/captopril supplementation in drinking water; however, the plasma nitrate level increased significantly in SHRSP5/Dmcr rats fed the HFC diet compared to that in SHRSP5/Dmcr and control WKY rats fed the SP diet. The level also increased significantly in SHRSP5/Dmcr rats fed the HFC diet with nitrite and captopril supplementation in drinking water for 8 weeks (Figure 8).

## 3. Discussion

NAFLD/NASH is a hepatic manifestation of metabolic syndrome characterized by obesity, glucose impairment, dyslipidemia, and hypertension. ROS generation caused by hepatic lipotoxicity and subsequent vascular endothelial dysfunction caused by inflammatory mediators could be a mechanism underlying NASH pathology, suggesting the possibility of impaired NO availability in this disease [16].

**Figure 9:**
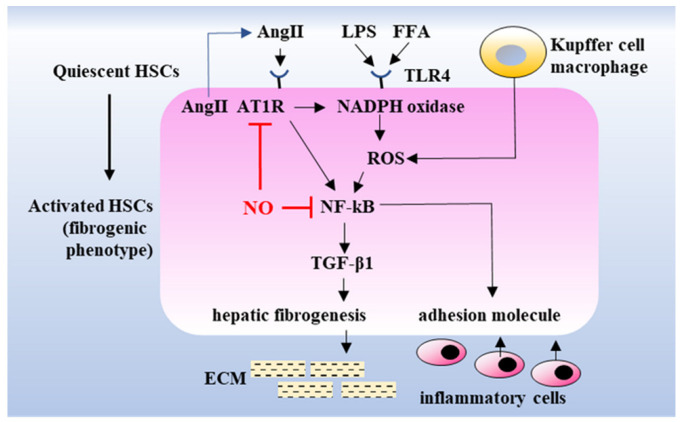
Proposed scheme of the molecular mechanism of NASH progression and its prevention by NO. HSCs are generally quiescent pericytes, whereas, upon stimulation by LPS and/or FFAs, they transdifferentiate into a fibrogenic phenotype that secretes ECM, leading to hepatic fibrosis via transcription factor-mediated signal transduction involving the Ang II-AT_1_R pathway, which also induces the expression of adhesion molecules, leading to further inflammatory processes. NO inhibits the AT_1_R and NF-κB-mediated upstream pathway of the inflammatory process. NASH, nonalcoholic steatohepatitis; HSCs, hepatic stellate cells; Ang II, angiotensin II; AT_1_R, angiotensin II type 1 receptor; LPS, lipopolysaccharide; FFAs, free fatty acids; NO, nitric oxide; NADPH, nicotinamide adenine dinucleotide phosphate; TLR4, Toll-like receptor 4; ROS, reactive oxygen species; NF-κB, nuclear factor-kappa B; TGF-β1, transforming growth factor-beta 1; ECM, extracellular matrix.

Lipotoxicity caused by accumulated lipids, along with the activation of the innate immune system, is a major causative factor in the development of NASH pathology. Cytotoxic lipid species, such as free cholesterol and free fatty acids released in the liver, activate inflammatory processes through toll-like receptors. This activates nuclear factor-kappa B (NF-κB) and NADPH oxidase, which stimulate hepatic stellate cells (HSCs) by inducing the expression of chemokines that cause fibrogenic HSC phenotype changes [17] and inflammatory cell adhesion to the hepatic vascular endothelium [18] (Figure 9). In the present study, although we observed that inflammatory cells infiltrated in the liver consisted of lymphocytes and macrophages in the rat NASH model (Figure S2), immunostaining revealed that the NADPH oxidase component p47phox and CD68 were mainly increased in the liver of the rat NASH model (Figure 4 and Figure 5). This suggests that the recruitment of activated monocytes and macrophages in the liver leads to ROS generation and activates inflammatory cascades, subsequently leading to liver fibrosis (Figure 5).

HSCs [19], resident mesenchymal cells, and major fibrogenic cells in the injured liver [20] are activated by extracellular signals from resident and inflammatory cells, such as Kupffer cells and macrophages, respectively. Angiotensin II type 1 receptor (AT_1_R) is expressed in activated HSCs, and angiotensin II (Ang II) enhances hepatic fibrosis through the production of transforming growth factor-beta 1 (TGF-β1). Previous studies have demonstrated that the blockade of Ang II synthesis and AT_1_R attenuates hepatic fibrosis in bile duct-ligated or CCl_4_-treated rats [21,22], suggesting that the Ang II-AT_1_R pathway could be involved in chronic inflammation and fibrosis in the liver (Figure 9) [23].

The therapeutic efficacy of angiotensin II type 1 receptor blockers (ARBs) has been demonstrated in patients with NASH [23]. In the present study, captopril treatment reduced liver fibrosis, possibly by inhibiting AT_1_R-mediated signaling to NF-κB activation, thereby inducing hepatic fibrogenesis (Figure 9). Yatabe et al. reported that ARB also lowered the mRNA levels of NADPH oxidase subunits, including p22phox and p47phox, in mesangial cells, suggesting the potential involvement of Ang II-AT_1_R-mediated signaling in NADPH oxidase activation [24]. Consistent with the findings of this report, the present study also showed that dietary nitrite supplementation decreased the p47phox immunohistochemical staining in the liver of the rat NASH model (Figure 4 and Figure 5).

Vascular endothelial function in SHRSP5/Dmcr rats is further impaired by HFC diet intake, which causes atherosclerosis and hypertension [8]. Therefore, increased aortic stiffness and concomitant hypertension contribute to cardiac remodeling owing to an increased LV afterload. During cardiac remodeling, hypertensive cardiomyocytes with adaptive hypertrophy develop maladaptive hypertrophy, characterized by the enlargement of the left ventricular chamber (eccentric hypertrophy) caused by excessive extracellular matrix degradation with disruption of cellular organization [25], which could cause myocyte lengthening and left ventricular chamber dilatation, as shown in our study (Figure 7). Although multiple signal transductions are involved in cardiac remodeling, the Ang II-AT_1_R pathway may play an important role in cardiac remodeling in NASH. Ang II mediates its effect by binding to AT_1_R present in vascular smooth muscle cells and myocytes, which leads to adverse cardiovascular effects, including NADPH oxidase-activated ROS generation and TGF-β-mediated cardiac remodeling and hypertrophy [26,27,28]. Clinical and experimental studies have reported significant benefits of the pharmacological blockade of the Ang II-AT_1_R pathway [29,30]. NO also exerts inhibitory effects on Ang II signaling activation by suppressing the activity of ACE by interacting with the zinc-coordinated active center of this enzyme [31]. In addition, NO inhibits the binding of transcription factors to the AT_1_R gene promoter via the *S*-nitrosylation [32,33,34,35] of NF-κB, which is linked to the upregulation of fibrogenic cytokines, such as TGF-β1, and subsequent myocardial and coronary arteriolar hypertrophy and fibrosis [36,37].

Consistent with LV dilatation and the restorative action of dietary nitrite in the rat NASH model (Figure 7), in a subgroup experiment, the mRNA level of BNP, a clinical marker of heart failure [15], decreased in the myocardium of HFC diet-fed SHRSP5/Dmcr rats subjected to dietary nitrite supplementation (500 mg of nitrite in drinking water for 4 weeks) (Figure S4). This may have occurred due to a reduction of the LV afterload and downregulation of Ang II-AT_1_R signaling in response to dietary nitrite supplementation.

We compared the therapeutic effect of captopril with that of dietary nitrite in the NASH rat model. Captopril inhibited the catalytic conversion of Ang I to Ang II, followed by the downregulation of subsequent AT_1_R-mediated signaling (Figure 9). The present study also showed that captopril and nitrite exert similar inhibitory effects on NASH pathology in the liver and heart, suggesting that the Ang II-AT_1_R pathway could be a common pathway inhibited by both nitrite and captopril, and the same pathway may be involved in the development of NASH pathology in the liver and heart. In addition, it is noteworthy that the plasma levels of nitrate, a stable end-product of NO, increased significantly in the rat NASH model treated with captopril, even without dietary nitrite supplementation (Figure 8). Captopril, along with its inhibitory effect on the Ang II-AT_1_R pathway, activates bradykinin-mediated eNOS expression by inhibiting bradykinin degradation [38]. This indicates that captopril may exert a protective effect on the endothelium by inhibiting the Ang II-AT_1_R pathway-mediated inflammatory process and may help preserve vascular endothelial function, subsequently inducing bradykinin-mediated NO generation [39]. 

In the present study, dietary nitrite and captopril could not significantly inhibit such a rapidly developing hepatic steatosis with increased plasma levels of AST/ALT (Figure S1) as observed in the SHRSP5/Dmcr rats fed the HFC diet (Figure 3). However, they prevented the subsequent inflammatory response, leading to macrophage accumulation and liver fibrosis. If the two-hit theory applies to NASH development [40], it could be suggested that dietary nitrite and captopril suppress the immune responses following the initiation of the AT_1_R and NF-κB mediated pathway (the second hit), rather than suppressing toxic lipid deposition in the liver (the first hit) in this rat NASH model.

Important issues related to the findings of the present study are the use of dietary nitrite instead of dietary nitrate, the doses of nitrite in drinking water, and the complications associated with dietary nitrite intake, such as methemoglobinemia and carcinogenicity, in clinical settings.

First, in humans, dietary nitrate is effectively concentrated in the saliva through active transport from circulation to the salivary glands. However, owing to species differences, rodents such as rats and mice do not exhibit active salivary nitrate concentration. Therefore, dietary nitrite, instead of nitrate, was used in this translational study [41]. Second, the quantity of nitrite consumed by the rats was approximately 1.34 mg per day per rat (for the 67 mg/L doses of nitrite (NO_2_^−^) in drinking water, based on the assumption that the rats would drink 20 mL of water per day). Nitrite (dose: 4.5 mg/kg BW/day, calculated based on a BW of 300 g of each rat) could be administered orally by increasing the consumption of nitrate/nitrite-rich foods and vegetables [28,42]. Third, although the nitrate and nitrite contents in food and drinking water have been limited by regulations in several countries based on their acute and chronic toxicities, such as methemoglobinemia and carcinogenicity, there are very few reports on methemoglobinemia in infants fed formula milk prepared with well water with bacterial contamination [43]. Chronic exposure to nitrate in food (e.g., through high red meat intake) has been linked to an increased risk of cancer [44], whereas the World Cancer Research Fund Continuous Update Report in 2015 reported no consistent epidemiological evidence of an increased risk of cancer in humans owing to nitrate consumption (e.g., through fruits and vegetables) [45].

This study primarily discusses the NO-mediated therapeutic effects in a rat NASH model based on the results of the primary experiment (supplementation of the NASH model with 100 mg/L nitrite and captopril in drinking water for 8 weeks). However, we have also included the results of supplementary subgroup experiments (supplementation of 500 mg/L nitrite in drinking water for 4 weeks, Figures S4 and S5), which were performed as a preliminary experiment to support the findings of the primary experiment and to provide a basis for future studies in this area of research.

In this study, dietary nitrite attenuated disease progression in a rat model of NASH with cardiovascular involvement and provided an alternative therapeutic option for NASH. In addition to treatment with pharmacological agents, such as ACE inhibitors or ARBs, lifestyle changes, such as increasing the consumption of vegetables and chewing better for greater saliva secretion [46], may help prevent NASH development.

## 4. Materials and Methods

### 4.1. Drug

Sodium nitrite and the ACE inhibitor captopril were purchased from Wako Pure Chemical Industries, Ltd. (Osaka, Japan).

### 4.2. Animals

Male WKY and SHRSP5/Dmcr rats (age: 9 weeks) were purchased from Japan SLC, Inc. (Hamamatsu, Japan). SHRSP5/Dmcr rats are established as parallel lines from outbred WKY rats [47,48]. All animal experiments were performed in compliance with the guidelines for animal experiments proposed by the Kinjo Gakuin University Animal Center. The study protocol was approved by the Committee on Ethics of Animal Experiments of the Kinjo Gakuin University Animal Center (approval numbers: 114/19/August/2015 and 165/19/August/2018).

The animals were acclimatized to the environment by providing access to distilled water and a standard diet (SP diet; Funabashi Farm, Chiba, Japan) for 7 days.

Two separate studies were performed using 10-week-old rats based on the experimental duration (8-week and 4-week groups). The 8-week group (primary group) was further divided into five groups comprising six animals each, depending on the diet, and with or without sodium nitrite and captopril supplementation in drinking water: (1) WKY + SP diet, (2) SHRSP5/Dmcr + SP diet, (3) SHRSP5/Dmcr + HFC diet, (4) SHRSP5/Dmcr + HFC diet + sodium nitrite (100 mg/L), and (5) SHRSP5/Dmcr + HFC diet + captopril (100 mg/L). The 4-week subgroup was further divided into three groups comprising six animals each, depending on the diet and sodium nitrite supplementation in drinking water: (1) SHRSP5/Dmcr + SP diet, (2) SHRSP5/Dmcr + HFC-diet, and (3) SHRSP5/Dmcr + HFC-diet + sodium nitrite (500 mg/L).

The control diet (SP control chow diet, 20.8% protein, 4.8% fat, 3.2% fiber, and 58.2% carbohydrate) (experimental details provided in Supplementary Information) and HFC diet (84% control diet, 12.5% palm oil, 2.5% cholesterol, and 1% colic acid) were purchased from Funabashi Farm (Chiba, Japan).

All rats were housed in a temperature- and light-controlled environment (temperature, 23 ± 2 °C; humidity, 55% ± 5%; light/dark cycle, 12 h) with access to a control diet and tap water ad libitum. At the end of the experiment, the rats were euthanized by the intraperitoneal administration of high-concentration pentobarbital (150 mg/kg; Somnopentyl^®^, Kyoritsu Seiyaku Ltd., Tokyo, Japan) Animal ethics, Cardiovasc Res. 2012. In addition, rodents, such as rats and mice, do not concentrate nitrates that are absorbed into saliva and circulate the body. Therefore, in this study, sodium nitrite was used instead of nitrate to reproduce the intestinal-saliva circulation pathway as in humans [41,49,50].

### 4.3. Arterial Blood Pressure

The rats were anesthetized using thiobutabarbital (Inactin^®^, 80 mg/kg ip, St. Louis, MO, USA), which can maintain a stable anesthetic effect for 3 to 4 h without suppressing the autonomic reflex (the depth of anesthesia was confirmed using the respiratory rate and stimulus reflex to the tail). Reportedly, actin exerts a limited effect on cardiovascular function (heart rate, blood pressure, and arterial pH) compared with other anesthetics [51]. A cannula (INTERMEDIC^TM^ PE-50tubing, Becton Dickinson and Company, Sparks, MD, USA) was placed in the femoral artery of the lower extremities to measure the arterial blood pressure while maintaining body temperature in an animal waterbed, and heparin (30 IU/mL) diluted with physiological saline was injected to prevent blood coagulation. Following this, it was connected to a Transducer (MLT0670, BP transducer, AD Instruments, Oxford, UK), the blood pressure was measured continuously for 10 min using the PowerLab^®^ system (AD Instruments, Oxford, UK), and the average value was calculated [28].

### 4.4. Histological Analysis

The heart and liver tissues were fixed in 10% (*w*/*v*) neutral buffered formalin and embedded in paraffin, and 4 μm-thick sections were prepared. The heart and liver tissues were subjected to HE staining and Masson’s trichrome staining, with the fibers stained blue. The liver tissues were stained with Sirius Red, with the fibers stained red.

HE-stained tissue in the entire heart area was imaged using a stereomicroscope (SZ61, Olympus, Tokyo, Japan). Using the captured images, the total area of the heart, myocardial area of the LV, lumen area of the LV, and ratio of the lumen area of the LV to the total area of the LV were calculated. Liver tissue fibrosis, liver tissue fatty area, and fibrosis around the blood vessels of the heart were imaged using an optical microscope equipped with a high-resolution video camera (CX41, Olympus). HE-stained liver tissue specimens were randomly photographed in ten fields at a 200× magnification using an optical microscope equipped with a high-resolution video camera. The ratio (%) of fatty area (white region unstained with HE-staining) to the total area of the captured image was calculated. Masson’s trichrome-stained heart tissue specimens were approximately ten coronary arteries with diameters of 50–300 μm were photographed from each heart tissue section at a magnification of 400×. The ratio (%) of the fibrous area (the adventitia) around the blood vessels to the total area of the blood vessels (intima, media, and adventitia) was calculated. Sirius Red-stained liver tissue specimens were randomly photographed in ten fields at a 100× magnification using an optical microscope equipped with a high-resolution video camera. The ratio (%) of fibrosis (red-colored region with Sirius Red staining) to the total area of the captured image was calculated. All images were analyzed using the ImageJ software (version 1.52, National Institutes of Health, USA).

### 4.5. Immunohistochemistry Analysis

The heart and liver tissues were fixed in 10% (*w*/*v*) neutral buffered formalin and embedded in paraffin, and 4 μm-thick sections were prepared. The sections were deparaffinized with xylene, and endogenous peroxidase activity was blocked with hydrogen peroxide. Next, the cells were washed with phosphate buffer and treated overnight with anti-CD68 (Abcam plc, Cambridge, UK) or anti-p47phox (Santa Cruz Biotechnology, Inc., Dallas, CA, USA) antibodies. They were then treated with the secondary antibody MAX-PO (MULTI) (Nichirei Biosciences Inc., Tokyo, Japan). Color was developed using the peroxidase substrate diaminobenzidine (ImmPACT TM DAB; Vector Laboratories, Inc., Burlingame, CA, USA) [52]. Immunostaining was quantified by randomly imaging CD68 (ten fields of liver tissue at 100×) or p47phox (20 fields of liver tissue at 400×) staining using an optical microscope equipped with a high-resolution video camera (CX41; Olympus, Tokyo, Japan). Next, the ratio (%) of the positively stained region (brown area) to the total area of the liver tissue was calculated. All images were analyzed using the ImageJ software (version 1.52, National Institutes of Health, New York, NY, USA).

### 4.6. RT-PCR

Heart tissue samples collected during dissection were immediately immersed in RNA stabilizing solution (RNA later^®^ solution; Ambion, Carlsbad, CA, USA) and stored at –30 °C until measurement. RNA was extracted from the heart tissues using ISOGEN II (Nippon Gene Co., Ltd., Tokyo, Japan). The extracted RNA was converted to cDNA using a cDNA synthesis kit (PrimeScript^®^ II; Takara Bio Inc., Shiga, Japan).

RNA was amplified by adding 1 μL of cDNA and a primer to the Gene RED PCR Mix (Nippon Gene Co., Ltd., Tokyo, Japan), and the reaction was performed in a thermal cycler (PC806, ASTEC, Fukuoka, Japan). Primers for the target mRNAs (ANP, BNP, and glyceraldehyde-3-phosphate dehydrogenase [GAPDH]) were synthesized by STAR-OLIGO (Rikaken Co., Ltd., Nagoya, Japan). The primers pairs were designed as follows: GAPDH: forward primer, 5′-CGGAGTCAACGGATTTGGTCGTAT-3′, reverse primer, 5′-AGCCTTCTCCATGGTGGTGAAGAC-3′ (300 bp, 32 cycles); ANP: forward primer, 5′-GGGCTCCTTCTCCATCACC-3′, reverse primer, 5′-CTCCAATCCTGTCAATCCTACC-3′(475 bp, 35 cycles;) BNP: forward primer, 5′-CAGCTCTCAAAGGACCAAGG-3′, reverse primer, 5′-AGAGCTGGGGAAAGAAGAGC-3′ (256 bp, 35 cycles). PCR amplification was conducted as follows: denaturation at 94 °C for 1 min, annealing of each GAPDH at 55 °C for 30 sec, ANP at 55 °C for 30 sec, BNP at 62 °C for 45 sec, and extension at 72 °C for 1 min [53,54]. The amplified mRNA sample was electrophoresed on a 2% agarose gel (TAE buffer: 0.04 M Tris-acetate, 0.001 M EDTA), stained with ethidium bromide, and photographed. The emission intensity of the band in the gel was quantified using the ImageJ software (v.1.52, National Institutes of Health), and the ratio of the target mRNA expression level to the GAPDH mRNA expression level was calculated.

### 4.7. Nitrite and Nitrate Concentration Measurement

Blood samples collected for dissection were immediately immersed in liquid nitrogen and stored at –80 °C until use. The samples were treated with plasma: methanol (1:1, volume/volume) to remove proteins and were then centrifuged (10,000× *g*, 5 min, room temperature). The nitrate and nitrite ion concentrations were measured using an HPLC system (ENO-20, Eicom, Kyoto, Japan) for NOx measurement based on the Griess method [55,56].

### 4.8. Plasma AST and ALT Concentration Measurement

Plasma AST and ALT levels were determined by SRL Inc. (Tokyo, Japan).

### 4.9. Statistical Analysis

All values are expressed as mean ± SE. Data were analyzed using one-way ANOVA, and differences among means were analyzed using the Tukey–Kramer multiple comparisons test. Statistical significance was set at *p* < 0.05.

## Data Availability

The data presented in this study are available on request from the corresponding author.

## Supplementary Materials

The following supporting information can be downloaded at: https://www.mdpi.com/article/10.3390/ijms23062931/s1.

## Abbreviations

SHRSP5/Dmcr: stroke-prone spontaneously hypertensive 5/Dmcr; NAFLD: nonalcoholic fatty liver disease; NASH: nonalcoholic steatohepatitis; HFC: high-fat and high-cholesterol; SP: stroke-prone; NADPH oxidase: nicotinamide adenine dinucleotide phosphate oxidase; ROS: reactive oxygen species; Ang-II: angiotensin II; AT1R: angiotensin II type 1 receptor; BNP: brain natriuretic peptide; ANP, atrial natriuretic peptide.
