# Supplementary material for: Beneficial Effects of Dietary Nitrite on a Model of Nonalcoholic Steatohepatitis Induced by High-Fat/High-Cholesterol Diets in SHRSP5/Dmcr Rats: A Preliminary Study
Source: Int J Mol Sci. 2022 Mar 8;23(6):2931. doi: 10.3390/ijms23062931 (PMC8951310; doi:10.3390/ijms23062931)
Supplement: Supplementary file 1 [file ijms-23-02931-s001.zip › ijms-1606203-supplementary.pdf]

Supplementary Figures

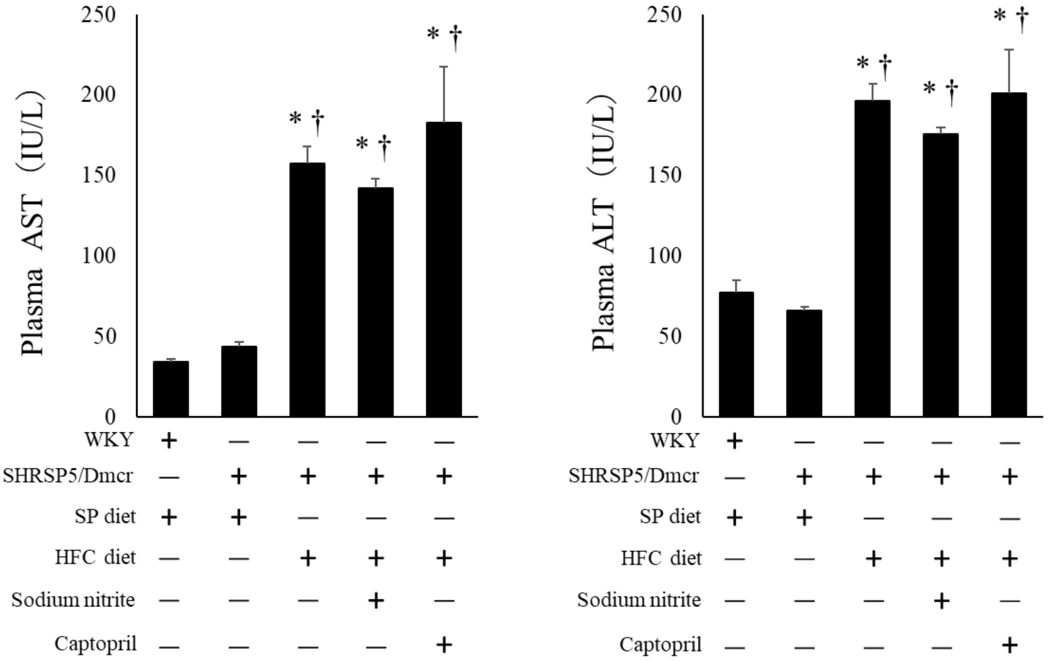

Figure S1: Effects of nitrite and captopril supplementation on plasma levels of AST and ALT in rat nonalcoholic steatohepatitis model.
Values represent mean ± SE (n=6), **P*<0.05 vs. WKY + SP diet group, †*P*<0.05 vs. SHRSP5/Dmcr + SP diet group.

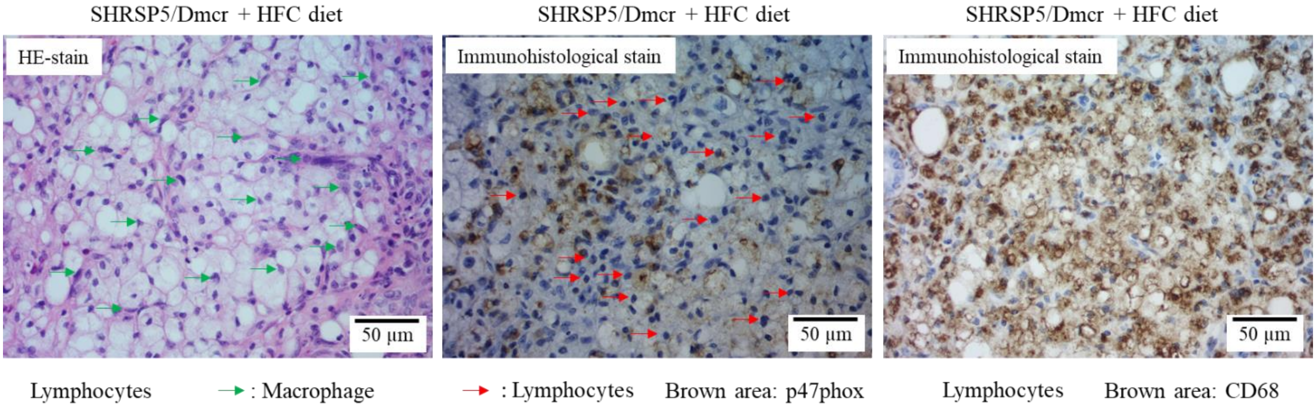

Figure S2: Inflammatory cells in the liver of rat nonalcoholic steatohepatitis model.
Inflammatory cell infiltration is composed of macrophages and lymphocytes (left: HE staining). Identification of inflammatory cells by immunostaining with the antibodies of p47phox (middle) and CD68 (right).

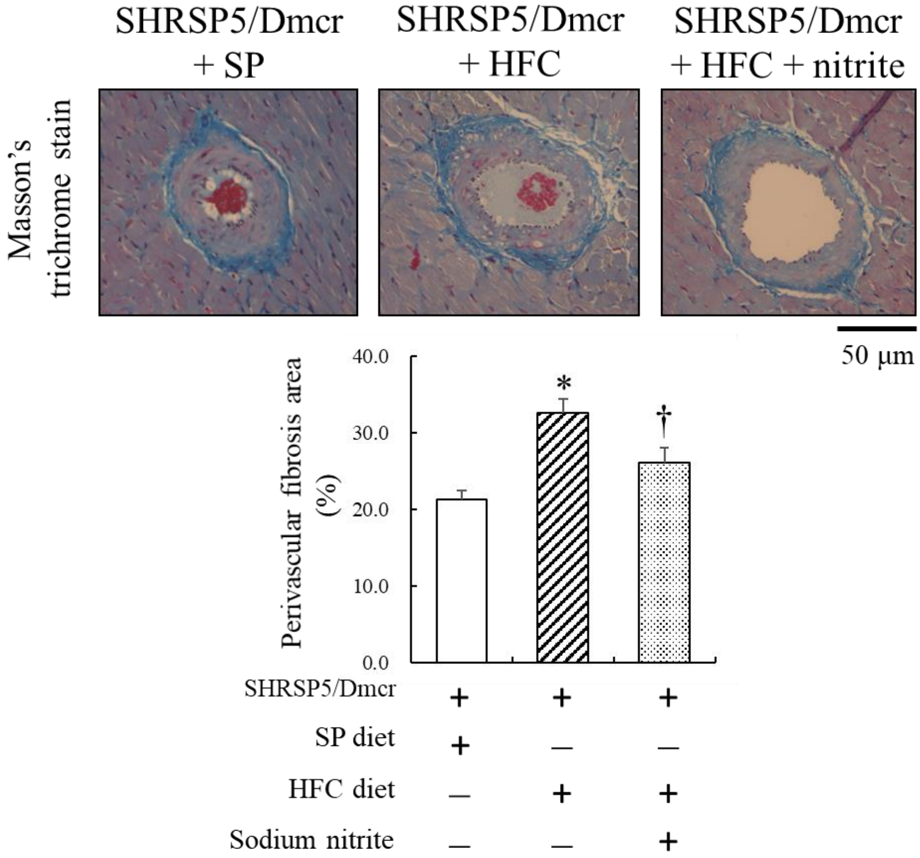

Figure S3: Histological assessment of coronary arterioles in rat nonalcoholic steatohepatitis model treated with/without nitrite.

SHRSP5/Dmcr rats were divided into three groups, each fed the SP diet, HFC diet, or HFC diet with nitrite supplementation in drinking water (500 mg/L) for 4 weeks.

Values represent mean ± SE (n=4), **P*<0.05, vs. the SHRSP5/Dmcr + SP diet group, †*P*<0.05, vs. the SHRSP5/Dmcr + HFC diet group.

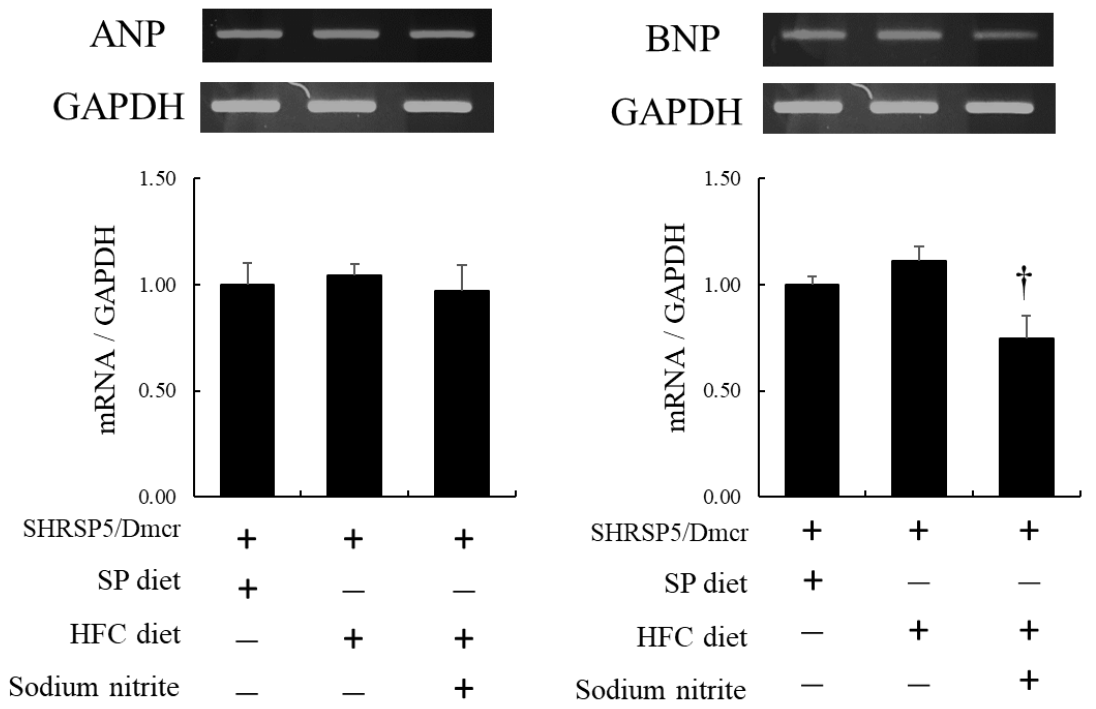

Figure S4: Myocardial transcription levels of ANP and BNP in the rat nonalcoholic steatohepatitis model treated with/without nitrite.

SHRSP5/Dmcr rats were divided into three groups, each fed the SP diet, HFC diet, or HFC diet with nitrite supplementation in drinking water (500 mg/L) for 4 weeks.

Values represent mean ± SE (n = 4), †P<0.05, vs. the SHRSP5/Dmcr + HFC diet group.

ANP, atrial natriuretic peptide; BNP, brain natriuretic peptide.
